# Developing CGMap: Characterizing Continuous Glucose Monitoring Data in Patients with Type 2 Diabetes

**DOI:** 10.3390/biomedicines13051080

**Published:** 2025-04-29

**Authors:** Shuzhen Bai, Chu Lin, Xiaoling Cai, Suiyuan Hu, Jing Wu, Ling Chen, Wenjia Yang, Linong Ji

**Affiliations:** Department of Endocrinology and Metabolism, Peking University People’s Hospital, Beijing 100044, China; 1810301226@pku.edu.cn (S.B.); chulin@bjmu.edu.cn (C.L.); 1510301223@pku.edu.cn (S.H.); cheryl.wu@163.com (J.W.); 13488692993@163.com (L.C.); ywjsunshine@126.com (W.Y.)

**Keywords:** type 2 diabetes, continuous glucose monitoring, diabetes management

## Abstract

**Objectives**: This study will characterize continuous glucose monitoring (CGM) data in patients with type 2 diabetes in China, and assess the relationship between CGM-derived indicators and diabetes-related clinical parameters. **Methods**: The data for this study were collected from a randomized trial in China (ChiCTR2000039424) from February 2020 to July 2022 in which patients wore a CGM device for 14 days. Glycemia risk index (GRI), coefficient of variation (CV), standard deviation (SD), mean amplitude of glycemic excursions (MAGE), time in range (TIR), time above range (TAR), time below range (TBR), and estimate glycated hemoglobin (eA1c) were analyzed. Ordinary least square linear regression and the Spearman method were used to test the relationship between CGM-derived indicators and diabetes-related clinical parameters. **Results**: In all, 528 patients with type 2 diabetes from a randomized controlled trial were analyzed. It was shown that CV, SD, and MAGE increased with age and diabetes duration, but decreased with an increase in body mass index. Higher fasting plasma glucose, higher baseline HbA1c, and higher insulin resistance levels were associated with higher GRI, SD, MAGE, TAR, and eA1c, and they were associated with lower TIR. In addition, higher HOMA-2β was associated with higher TIR and TBR, and with lower TAR and eA1c. Hemoglobin had positive correlations to SD, TAR, and eA1c. **Conclusions**: It was found that glucose variability increased with age and the duration of diabetes. However, glucose variability decreased with increased BMI. Meanwhile, greater glycemic variability was associated with worse islet function, higher baseline glucose level, and higher hemoglobin.

## 1. Introduction

It is well known that glucose monitoring is important for diabetes care. With the glucose monitoring technology evolving, continuous glucose monitoring (CGM) appeared. CGM is performed by devices that estimate blood glucose levels by detecting the glucose concentration in the interstitial fluid between cells [1]. Although CGM is more expensive than SMBG, it has beenshown that compared with the use of self-monitoring blood glucose (SMBG), the use of CGM significantly reduces the levels of hemoglobin A1c (HbA1c), body weight, calorie intake, and quality-adjusted life years (QALYs) [2,3,4,5]. CGM is the preferred glucose monitoring method in the most recent American Diabetes Association (ADA) guideline for patients with insulin-requiring diabetes [6]. Therefore, we are in the era of continuous glucose monitoring (CGM).

The use of CGM is individualized. For the definitive use of CGM, it is important to understand the relationships between CGM-derived indicators and diabetes-related clinical parameters, which is also meaningful for the clinical practice of diabetes management. Meanwhile, a study showed a CGMap of more than 7000 non-diabetes adults, which provided reference values of key CGM-derived clinical measures [7]. However, in patients with type 2 diabetes (T2D), similar CGMaps are scarce. As for the indicators reflecting glucose variability using CGM, it was found that mean amplitude of glycemic excursions (MAGE) or standard deviation (SD) were positively correlated with age and HbA1c [8,9], while others indicated negative correlations [10,11]. Opposite reports on the relationship between MAGE and body mass index (BMI) were also found [8,10]. In addition, limited studies reported the correlations between HbA1c levels and the coefficient of variation (CV) or glycemia risk index (GRI) [12].

As the relationships between MAGE, SD, CV, or other CGM-derived indicators and age, BMI, as well as baseline HbA1c are controversial, the relationships between these CGM-derived indicators and other parameters such as blood pressure, liver function, or kidney function, etc., have rarely been well discussed.

In this study, we used clinical data derived from a randomized clinical trial (RCT) in patients with T2D, by developing a CGMap of T2D, to explore the relationship between CGM-derived indicators and demographic and clinical parameters in patients with T2D.

## 2. Materials and Methods

### 2.1. Participants

The data for this study were collected from a randomized, open-label, active-controlled, parallel-group trial conducted at 15 centers in China from February 2020 to July 2022 (ChiCTR2000039424) [13]. Participants enrolled in the study were those with T2D aged 18–65 years, with a BMI of 19–40 kg/m^2^, and a HbA1c level between 6.5 and 9.0%. Patients received metformin combined with either acarbose or sitagliptin, and after the first 14-day treatment period, patients wore an rtCGM and then continued with the next 14-day treatment period. In all, 528 patients with T2D from this randomized controlled trial were analyzed.

### 2.2. CGM-Derived Indicators

Variability measures:

(1) GRI was calculated as (3.0 × VLow) + (2.4 × Low) + (1.6 × VHigh) + (0.8 × High), where VLow represents very-low-glucose hypoglycemia (<3.0 mmol/L), Low represents low-glucose hypoglycemia (≥3.0 and <3.9 mmol/L), High represents high-glucose hyperglycemia (>10.0 and ≤13.9 mmol/L), and VHigh represents very-high-glucose hyperglycemia (>13.9 mmol/L) [14].
(2) SD was calculated as the square root of the average squared difference between each glucose datum and the mean. $SD=\frac{\sqrt{\sum {\left(X-\bar{x}\right)}^{2}}}{n-1}$, where *X* = each glucose datum; $\bar{x}$ = mean glucose; *n* = number of values in the sample.
(3) CV was calculated as SD divided by the mean glucose value multiplied by 100.
(4) MAGE was calculated as the average of all blood glucose (BG) increases or decreases that are >1 standard deviation of all BG measures [15].

Glucose management measures:

(1) Time in range (TIR) was calculated as the number of mean glucose values at 3.9–10.0 mmol/L divided by the number of total values multiplied by 100.
(2) Time below range (TBR) (below target level < 3.9 mmol/L) was calculated as the number of mean glucose values below 3.9 mmol/L divided by the number of total values multiplied by 100.
(3) TBR (below target level < 3.0 mmol/L) was calculated as the number of mean glucose values below 3.0 mmol/L divided by the number of total values multiplied by 100.
(4) Time above range (TAR) (above target level > 10.0 mmol/L) was calculated as the number of mean glucose values above 10.0 mmol/L divided by the number of total values multiplied by 100.
(5) TAR (above target level > 13.9 mmol/L) was calculated as the number of mean glucose values below 3.9 mmol/L divided by the number of total values multiplied by 100.
(6) Estimate hemoglobin A1c (eA1c) was calculated as (46.7 + mean glucose) divided by the 28.7.

### 2.3. Clinical Parameters

We focused on 19 clinical parameters of interest that were related to glycemic variability as the following 8 categories:

(1) Demographic parameters: age, sex, and the duration of diabetes.
(2) Baseline blood glucose parameters: HbA1c and fasting plasma glucose (FPG).
(3) Islet function parameters: fasting insulin, fasting C-peptide, and homeostasis model assessment β-cell function (HOMA2-β). HOMA2-β was calculated by the HOMA2 calculator [16].
(4) Insulin resistance parameters: homeostasis model assessment insulin resistance (HOMA IR), which was calculated as (FPG × fasting insulin)/22.5.
(5) Physical examination parameters: BMI, systemic blood pressure (SBP), and diastolic blood pressure (DBP).
(6) Renal-related parameters: serum creatinine (SCr) and estimated glomerular filtration rate (eGFR).
(7) Liver-related parameters: alanine aminotransferase (ALT), aspartate aminotransferase (AST), and ALT/AST.
(8) Blood routine parameters: hemoglobin (HB) and hematocrit (HCT).

### 2.4. Statistical Analyses

Continuous variables will be reported as median ± interquartile spacing. Categorical variables will be presented as absolute values with percentages. Differences between groups will be compared using the Kruskal–Wallis rank sum test. The Mann–Whitney test was used to test whether there were gender differences in CGM-derived indicators. The Spearman method was used to test the correlation between CGM-derived indicators and other diabetes-related clinical parameters. Ordinary least square (OLS) linear regression was used for regression analysis to describe the relationship between the CGM-derived indicator and diabetes-related clinical parameters.

A two-sided *p*-value < 0.05 was considered statistically significant in all statistical tests. Statistical analysis was conducted using SPSS 23.0 and Python 3.11.3.

## 3. Results

### 3.1. Patient Characteristics

Among these 528 patients, 55.1% of them were male. The median (interquartile spacing) age was 56 (13) years old, baseline BMI was 25.35 (3.98) kg/m^2^, and baseline HbA1c was 7.10 (1.06)%. The median (interquartile spacing) GRI level was 12.25 (18.11), CV was 22.32 (6.57)%, SD was 1.61 (0.60) mmol/L, MAGE was 4.36 (1.73) mmol/L, TIR was 89.16 (16.09)%, TBR (below 3.0 mmol/L) was 0.00 (0.31)%, TBR (below 3.9 mmol/L) was 0.52 (2.68)%, TAR (above 10.0 mmol/L) was 7.37 (14.84)%, TAR (above 13.9 mmol/L) was 0.15 (1.44)%, and eA1c was 6.00 (1.10)%.

### 3.2. CGMap Characterized by Age

**Figure 1:**
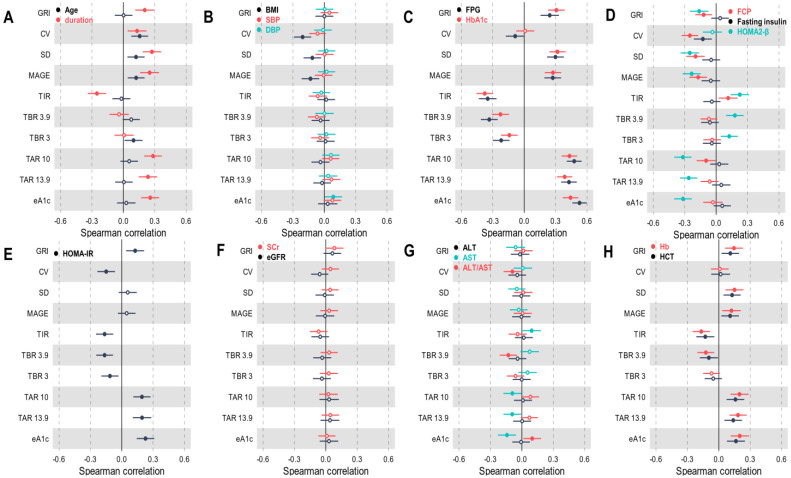
Correlation of CGM-derived measures with clinical measures. Spearman correlation between CGM-derived indicators and other diabetes-related clinical parameters. The dots represent the correlation coefficients. (**A**) Demographic parameters; (**B**) physical examination parameters; (**C**) baseline blood glucose parameters; (**D**) islet function parameters; (**E**) insulin resistance parameters; (**F**) liver-related parameters; (**G**) renal-related parameters; (**H**) blood routine parameters. Dots present the correlation coefficient, with lines marking the 95% CIs.

**Figure 2:**
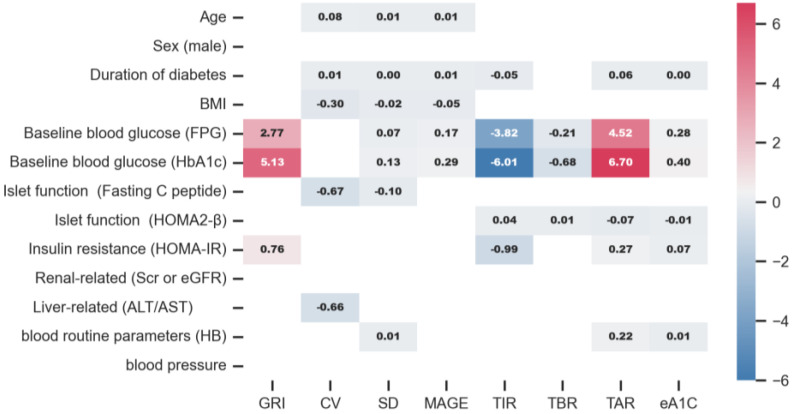
Heatmap of the slope of linear regression between CGM-derived indicators and clinical parameters. Ordinary least square linear regression between the CGM-derived indicator and diabetes-related clinical parameters. Clinical parameters were independent variables.

**Figure 3:**
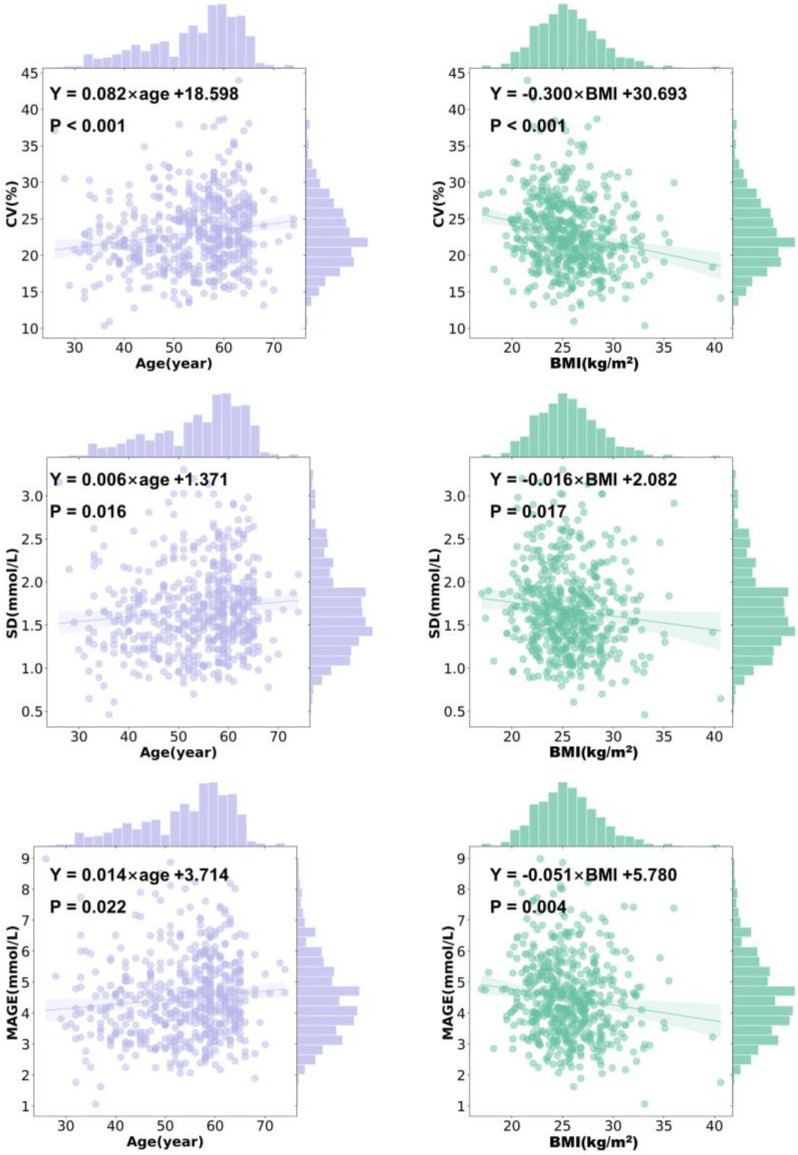
The features of CGM characterized by age and BMI. Ordinary least square linear regression between the CGM-derived indicator and age and BMI, respectively. Purple, age; green, BMI. Dots show data points, lines show linear regression, and shadows show 95% CI. Clinical parameters were independent variables. The regression equation is shown in black.

An older age was associated with a higher CV (r = 0.156, *p* < 0.001), SD (r = 0.121, *p* = 0.005), a higher MAGE (r = 0.122, *p* = 0.005), and a higher level of TBR (below 3.0 mmol/L) (r = 0.097, *p* = 0.026) (Figure 1A). It was found that CV increased by 0.082% (95% CI 0.036 to 0.128, *p* <0.001) per year old, and MAGE increased by 0.014 mmol/L (95% CI 0.002 to 0.026, *p* = 0.022) per year old in patients with T2D (Figure 2 and Figure 3). Similarly, SD slightly rose with age in all patients (β = 0.006, 95% CI 0.001 to 0.010, *p* = 0.016) (Figure 2 and Figure 3). However, indicators including GRI, TIR, TAR, TBR, and eA1c did not seem to correlate with age in patients with T2D.

### 3.3. CGMap Characterized by the Sex

In the patients with T2D included, males (median 7.10%) and females (median 7.10%) had similar HbA1c levels (*p* = 0.162). Compared with female patients with T2D, GRI (Z = −3.341, *p* = 0.001), TAR above 10 mmol/L (Z = −2.086, *p* = 0.037), and TAR above 13.9 mmol/L (Z = −2.408, *p* = 0.016) were higher in male patients with T2D. There was no significant difference in other CGM-derived measures between the sexes (Table A1).

### 3.4. CGMap Characterized by Duration of Diabetes

**Figure 4:**
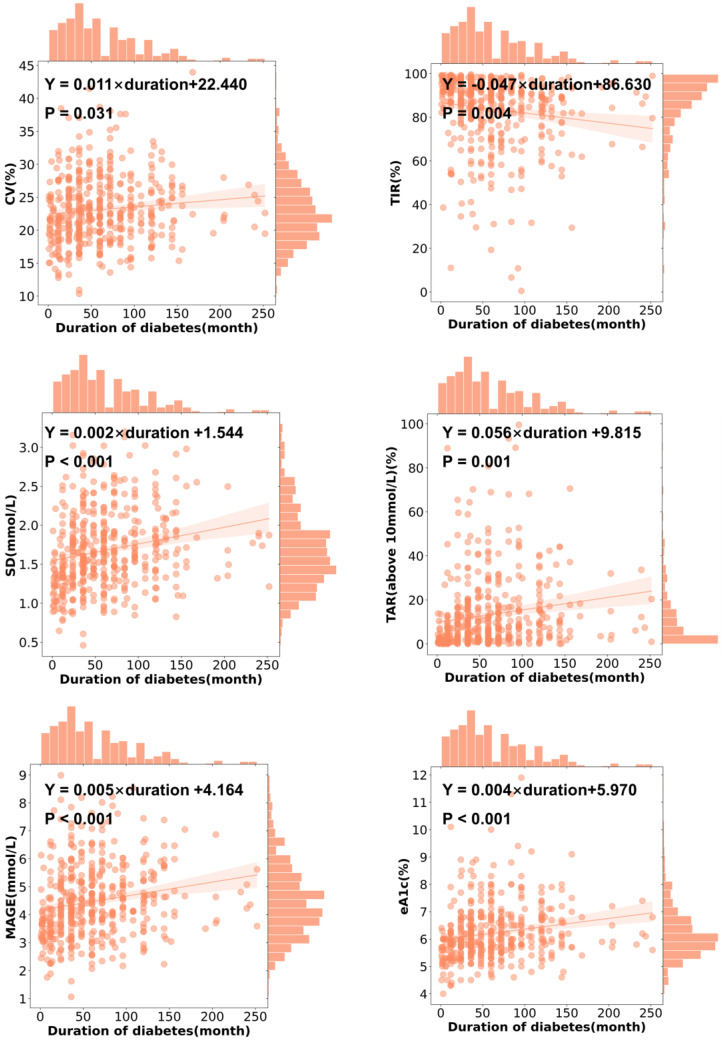
The features of CGM characterized by duration of diabetes. Ordinary least square linear regression between the CGM-derived indicator and diabetes duration. Clinical parameters were independent variables. Dots show data points, lines show linear regression, and shadows show 95% CI. The regression equation is shown in black.

A longer duration of diabetes was associated with a higher GRI (r = 0.204, *p* < 0.001), a higher CV (r = 0.131, *p* = 0.005), a higher SD (r = 0.273, *p* < 0.001), a higher MAGE (r = 0.251, *p* < 0.001), a higher level of TAR above 10 mmol/L (r = 0.283, *p* < 0.001), a higher level of TAR above 13.9 mmol/L (r = 0.235, *p* < 0.001), and a higher level of eA1c (r = 0.257, *p* < 0.001), but a lower level of TIR (r = −0.253, *p* < 0.001) (Figure 1A). With every year increase in the duration of diabetes, CV increased by 0.011% (95% CI 0.001 to 0.021, *p* = 0.031) and SD increased by 0.002 mmol/L (95% CI 0.001 to 0.003, *p* < 0.001) in patients with T2D (Figure 2 and Figure 4). Similarly, MAGE rose slightly as the duration of diabetes increased (β = 0.005 mmol/L, 95% CI 0.002 to 0.008, *p* < 0.001) in patients with T2D. The increase in the duration of diabetes was also associated with a higher level of eA1c (β = 0.004, 95% CI 0.002 to 0.006, *p* < 0.001). Correspondingly, as the duration of diabetes prolonged, the level of TIR (β = −0.047, 95% CI −0.079 to −0.015, *p* = 0.004) decreased while the level of TAR above 10 mmol/L (β = 0.056, 95% CI 0.024 to 0.088, *p* = 0.001) increased in patients with T2D (Figure 2 and Figure 4).

### 3.5. CGMap Characterized by BMI

BMI was positively and significantly correlated to CV (r = −0.207, *p* < 0.001), SD (r = −0.114, *p* = 0.002), and MAGE (r = −0.132, *p* = 0.001) (Figure 1B). With every 1 kg/m^2^ increase in BMI in patients with T2D, CV decreased by 0.300% (95% CI −0.429 to −0.170, *p* < 0.001), SD decreased by 0.016 mmol/L (95% CI −0.029 to −0.003, *p* = 0.017), and MAGE decreased by 0.051% (95% CI −0.085 to −0.016, *p* = 0.004). However, in patients with T2D, GRI, eA1c, TIR, TAR, and TBR were not associated with BMI (Figure 2 and Figure 3).

### 3.6. CGMap Characterized by Blood Glucose Level

**Figure A1:**
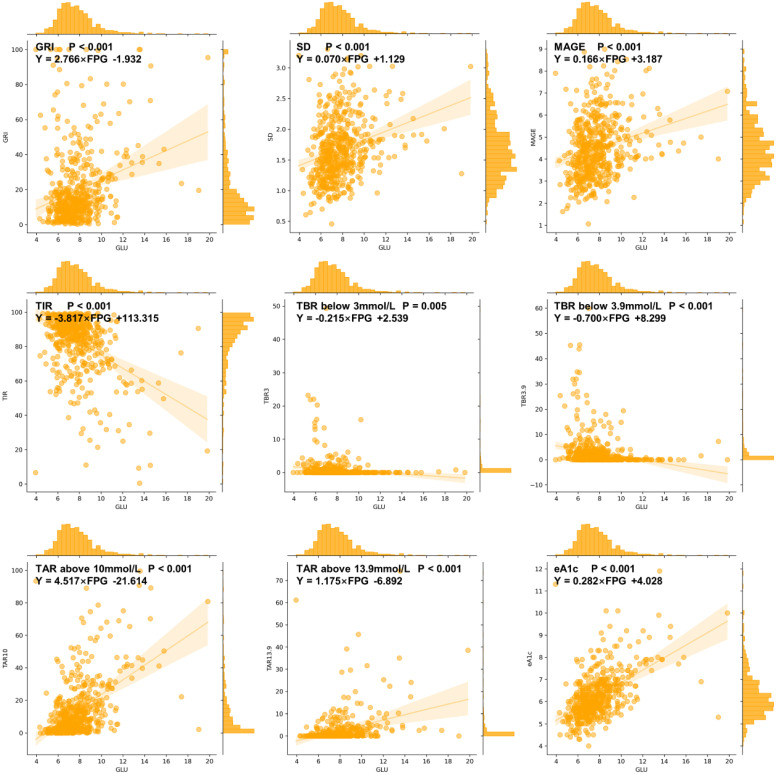
The features of CGM characterized by FPG. Ordinary least square linear regression between the CGM-derived indicator and FPG. Clinical parameters were independent variables. Dots show data points, lines show linear regression, and shadows show 95% CI. The regression equation is shown in black.

A higher baseline FPG was associated with a higher GRI (r = 0.244, *p* < 0.001), SD (r = 0.299, *p* < 0.001), a higher MAGE (r = 0.273, *p* < 0.001), a higher level of TAR above 10 mmol/L (r = 0.478, *p* < 0.001), a higher level of TAR above 13.9 mmol/L (r = 0.428, *p* < 0.001), and a higher level of eA1c (r = 0.529, *p* < 0.001), but a lower CV (r = −0.086, *p* = 0.047), a lower level of TIR (r = −0.349, *p* < 0.001), a lower level of TBR below 3.9 mmol/L (r = −0.334, *p* < 0.001), and a lower level of TBR below 3 mmol/L (r = −0.220, *p* < 0.001) (Figure 1C). For patients with T2D, every 1 mmol/L increase in FPG was associated with a 2.766% (95% CI 1.875 to 3.658, *p* < 0.001) increase in GRI, a 0.070 mmol/L (95% CI 0.049 to 0.091, *p* < 0.001) increase in SD, a 0.166% (95% CI 0.109 to 0.223, *p* < 0.001) increase in MAGE, a 4.517% (95% CI 3.873 to 5.162, *p* < 0.001) increase in TAR above 10 mmol/L, a 1.175% (95% CI 0.903 to 1.447, *p* < 0.001) increase in TAR above 13.9 mmol/L, and a 0.282% (95% CI 0.244 to 0.320, *p* < 0.001) increase in eA1c. However, when FPG increased by 1 mmol/L, the TIR decreased by 3.817% (95% CI −4.485 to −3.148, *p* < 0.001), the TBR below 3 mmol/L decreased by 0.215% (95% CI −0.363 to −0.067, *p* = 0.005), and the TBR below 3.9 mmol/L reduced by 0.700% (95% CI −0.982 to −0.419, *p* < 0.001) in patients with T2D (Figure 2 and Figure A1).

**Figure 5:**
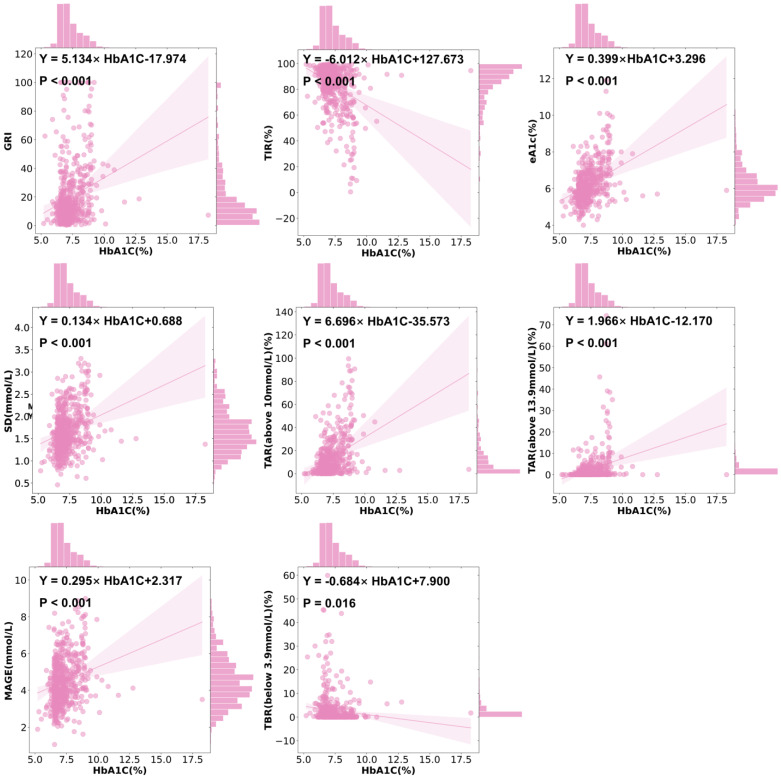
The features of CGM characterized by HbA1c. Ordinary least square linear regression between the CGM-derived indicator and HbA1c. Clinical parameters were independent variables. Dots show data points, lines show linear regression, and shadows show 95% CI. The regression equation is shown in black.

A higher HbA1c was associated with a higher GRI (r = 0.308, *p* < 0.001), SD (r = 0.318, *p* < 0.001), a higher MAGE (r = 0.275, *p* < 0.001), a higher level of TAR above 10 mmol/L (r = 0.433, *p* < 0.001), a higher level of TAR above 13.9 mmol/L (r = 0.387, *p* < 0.001), and a higher level of eA1c (r = 0.443, *p* < 0.001), but a lower level of TIR (r = −0.377, *p* < 0.001), a lower level of TBR below 3.9 mmol/L (r = −0.227, *p* < 0.001), and a lower level of TBR below 3 mmol/L (r = −0.142, *p* = 0.001) (Figure 1C). The univariate linear regression of HbA1c and CGM-derived indicators showed similar patterns to FPG (Figure 2 and Figure 5).

### 3.7. CGMap Characterized by Islet Function and Insulin Resistance

**Figure A2:**
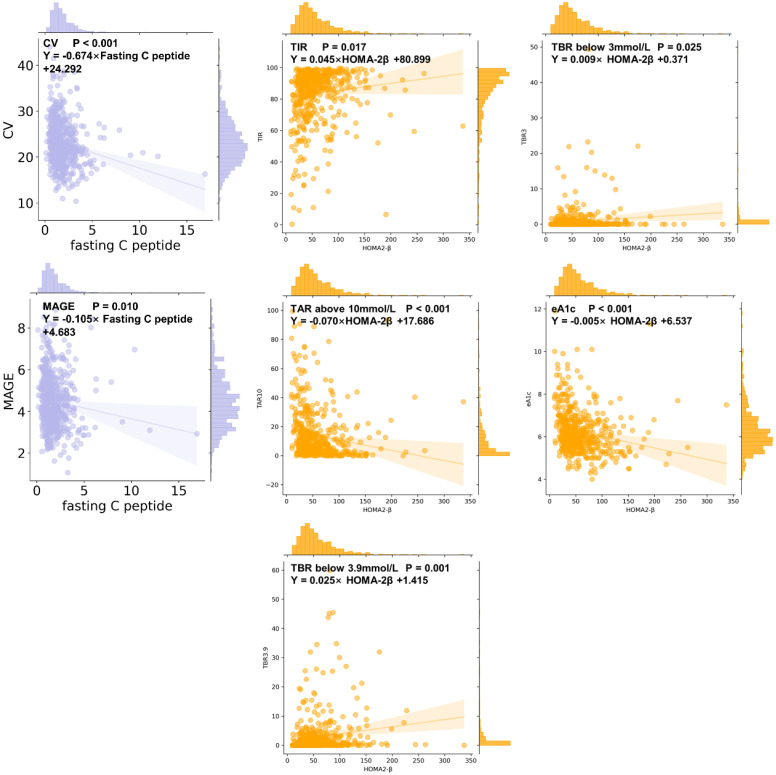
The features of CGM characterized by fasting insulin and HOMA2-β. Ordinary least square linear regression between the CGM-derived indicator and fasting insulin and HOMA2-β. Clinical parameters were independent variables. Purple, fasting C peptide; orange, HOMA2-β. Dots show data points, lines show linear regression, and shadows show 95% CI. The regression equation is shown in black.

It was found that a higher fasting insulin was associated with a higher CV (r = −0.127, *p* = 0.004) (Figure 1D). Meanwhile, fasting C-peptide was conversely associated with GRI (r = −0.119, *p* = 0.006), CV (r = −0.251, *p* < 0.001), SD (r = −0.197, *p* < 0.001), MAGE (r = −0.172, *p* < 0.001), TAR above 10 mmol/L (r = −0.096, *p* = 0.028) (Figure 1D). However, higher levels of fasting C-peptide were associated with increased levels of TIR (r = 0.115, *p* = 0.009) (Figure 1D). With every 1 nmol/L increase in fasting C-peptide in patients with T2D, CV was decreased by 0.674% (95% CI −0.970 to −0.378, *p* < 0.001) and MAGE was decreased by 0.105% (95% CI −0.184 to −0.025, *p* = 0.010) (Figure 2 and Figure A2).

Similarly to fasting C-peptide, a higher HOMA2-β was also associated with a lower GRI (r = −0.162, *p* < 0.001), a lower SD (r = −0.252, *p* < 0.001), a lower MAGE (r = −0.234, *p* < 0.001), a lower level of TAR above 10 mmol/L (r = −0.318, *p* < 0.001), a lower level of TAR above 13.9 mmol/L (r = −0.262, *p* < 0.001), and a lower level of eA1c (r = −0.317, *p* < 0.001), but a higher level of TIR (r = 0.226, *p* < 0.001), a higher level of TBR below 3 mmol/L (r = 0.125, *p* = 0.004), and a higher level of TBR below 3.9 mmol/L (r = 0.179, *p* < 0.001) (Figure 1D). For patients with T2D, every one increase in HOMA2-β was associated with a 0.045% (95% CI 0.008 to 0.083, *p* = 0.017) increase in TIR, a 0.009% (95% CI 0.001 to 0.016, *p* = 0.025) increase in TBR below 3 mmol/L, a 0.025% (95% CI 0.010 to 0.039, *p* = 0.001) increase in TBR below 3.9 mmol/L. Every one increase in HOMA2-β was associated with a 0.070% (95% CI −0.107 to −0.033, *p* < 0.001) decrease in TAR above 10 mmol/L, and a 0.005% (95% CI −0.008 to −0.003, *p* < 0.001) decrease in eA1c (Figure 2 and Figure A2).

**Figure A3:**
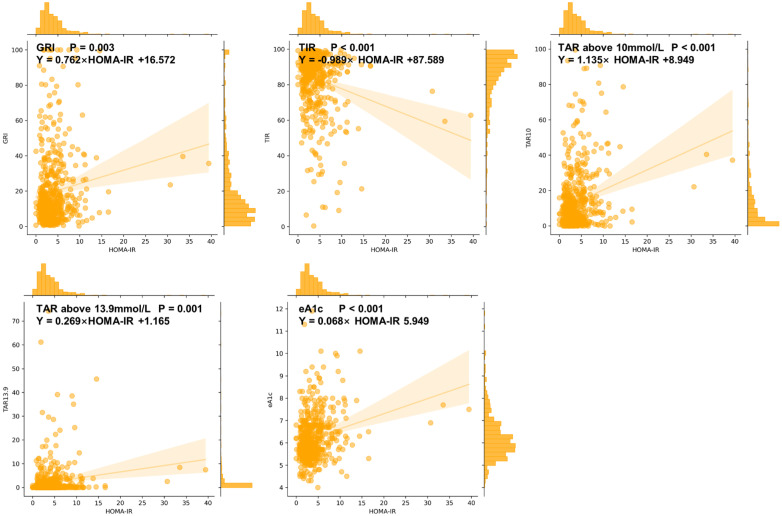
The features of CGM characterized by HOMA-IR. Ordinary least square linear regression between the CGM-derived indicator and HOMA-IR. Clinical parameters were independent variables. Dots show data points, lines show linear regression, and shadows show 95% CI. The regression equation is shown in black.

Concerning insulin resistance, a higher HOMA-IR was associated with a lower CV (r = −0.149, *p* = 0.001), a lower level of TIR (r = −0.164, *p* < 0.001), a lower level of TBR below 3 mmol/L (r = −0.113, *p* = 0.010), and a lower level of TBR below 3.9 mmol/L (r = −0.164, *p* < 0.001), but a higher GRI (r = 0.128, *p* = 0.003), a higher level of TAR above 10 mmol/L (r = 0.193, *p* < 0.001), a higher level of TAR above 13.9 mmol/L (r = 0.194, *p* < 0.001), and a higher level of eA1c (r = 0.227, *p* < 0.001) (Figure 1E). GRI increased by 0.762 mmol/L (95% CI 0.253 to 1.270, *p* < 0.001) with every increase in HOMA-IR in patients with T2D. TIR decreased by 0.989% (95% CI −1.394 to −0.584, *p* < 0.001), TAR above 10 mmol/L increased by 1.135% (95% CI 0.729 to 1.541, *p* < 0.001), TAR above 13.9 mmol/L increased by 0.269% (95% CI 0.109 to 0.428, *p* = 0.001), and eA1c increased by 0.068% (95% CI 0.043 to 0.092, *p* < 0.001) with every increase in HOMA-IR in patients with T2D (Figure 2 and Figure A3).

### 3.8. CGMap Characterized by Renal and Liver Function

Renal-related indicators including SCr and eGFR did not seem to be associated with any CGM-derived measures (Figure 1F).

**Figure A4:**
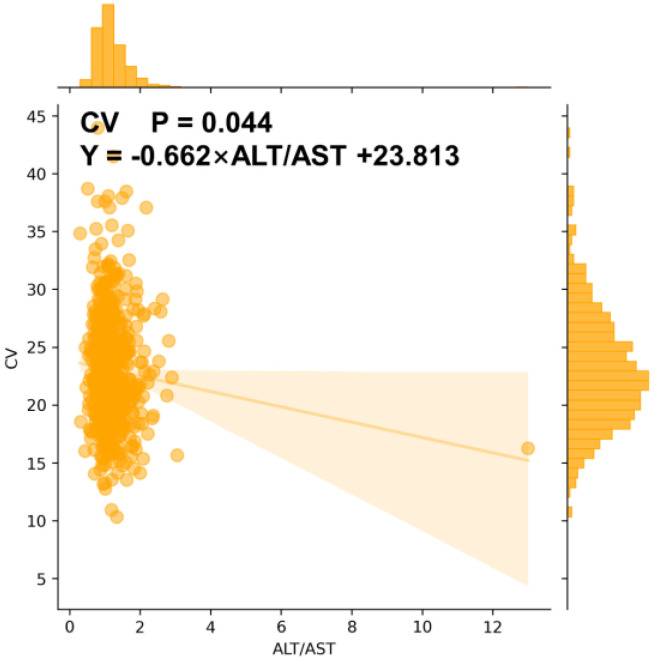
The features of CGM characterized by ALT/AST. Ordinary least square linear regression between the CGM-derived indicator and ALT/AST. Clinical parameters were independent variables. Dots show data points, lines show linear regression, and shadows show 95% CI. The regression equation is shown in black.

It was found that higher AST was associated with lower TAR 10 mmol/L (r = −0.090, *p* = 0.038), TAR 13.9 mmol/L (r = −0.092, *p* = 0.035), and eA1c (r = −0.142, *p* = 0.001), but it was associated with a higher TIR (r = 0.094, *p* = 0.032). Higher ALT/AST was associated with lower CV (r = −0.090, *p* = 0.040), and TBR below 3.9 mmol/L (r = −0.129, *p* = 0.003), but higher ALT/AST was associated with higher eA1c (r = 0.099, *p* = 0.024). ALT was uncorrelated with all the CGM-derived indicators (Figure 1G). Furthermore, as seen through univariate linear regression, CV reduced slightly as ALT/AST increased (β = −0.662, 95% CI −1.308 to −0.017, *p* = 0.044) in patients with T2D (Figure 2 and Figure A4).

### 3.9. CGMap Characterized by Blood Routine Parameters

**Figure A5:**
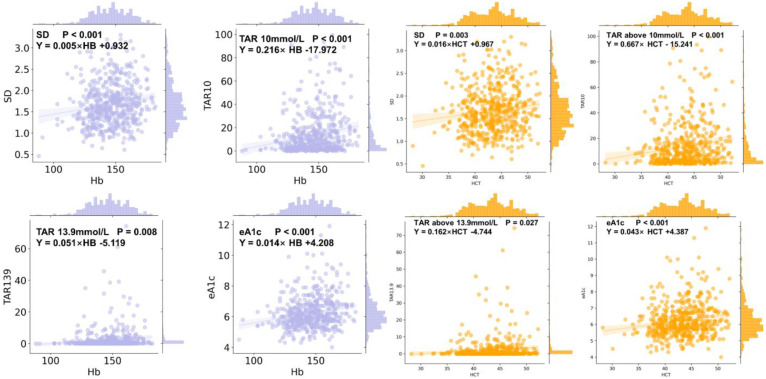
The features of CGM characterized by hemoglobin and hematocrit. Ordinary least square linear regression between the CGM-derived indicator and hemoglobin and hematocrit. Clinical parameters were independent variables. Purple, hemoglobin; orange, hematocrit. Dots show data points, lines show linear regression, and shadows show 95% CI. The regression equation is shown in black.

It was found that higher hemoglobin was correlated with higher GRI (r = 0.145, *p* = 0.001), SD (r = 0.150, *p* = 0.001), MAGE (r = 0.121, *p* = 0.006), TAR above 10 mmol/L (r = 0.198, *p* < 0.001), TAR above 13.9 mmol/L (r = 0.184, *p* < 0.001), and eA1c (r = 0.200, *p* < 0.001) (Figure 1H). Higher hemoglobin was correlated with lower TIR (r = −0.168, *p* < 0.001), and TBR below 3.9 mmol/L (r = −0.124, *p* = 0.004) (Figure 1H). In patients with T2D, every 1 g/L increase in HB was associated with a 0.005 mmol/L (95% CI 0.002 to 0.008, *p* < 0.001) increase in SD, a 0.216% (95% CI 0.120 to 0.313, *p* < 0.001) increase in TAR above 10 mmol/L, a 0.051% (95% CI 0.013 to 0.088, *p* = 0.008) increase in TAR above 13.9 mmol/L, and a 0.014% (95% CI 0.008 to 0.020, *p* < 0.001) increase in eA1c (Figure 2 and Figure 5). The univariate linear regression of HCT and CGM-derived indicators showed similar patterns to HB (Figure 2 and Figure A5).

### 3.10. CGMap and Blood Pressure

Higher DBP was associated with a higher eA1c (r = 0.087, *p* = 0.047) (Figure 1B). However, no significant association between CGM-derived indicators and blood pressure was found by regression analysis.

## 4. Discussion

Based on a post hoc analysis of the CGM data from an RCT which enrolled patients with T2D in China who wore an rtCGM for 14 days, we found that the trend of blood glucose variability changes with age and BMI in patients with T2D. Based on the CGM data, we also found that higher baseline blood glucose levels, lower B-cell function, and more hemoglobin were associated with greater blood glucose variability. We first detailed the results of CGMap and indicators of T2D. It is significant for future individualized and refined guidance of glucose monitoring and clinical diagnosis and treatment of T2D.

Our study found that glucose variability increased with the age of patients. It was shown that older age is associated with higher CV, SD, MAGE, and TBR. Similarly, in non-diabetes patients, blood glucose variability also increases with age [7]. Some studies have also found that TIR and glycemic variability worsen with age in Japanese patients with type 2 diabetes [17]. This is possibly due to the long course of the disease and the setting of high target glycemic levels in patients with advanced diabetes complications. It could also be related to the gradual decline of pancreatic function with age and the increase in insulin resistance [18]. Therefore, more attention should be paid to blood glucose monitoring and management for older patients.

According to this study, we found that GRI and TAR are higher in males compared with females. Previous research also found that, in a healthy population, the coefficient of variation in fasting blood glucose level is higher in males than in females, indicating a greater fluctuation of blood glucose in males [19]. Previous animal studies showed that estrogen has a protective effect on islet function, and taking estrogen in male rats could prevent isoxene-induced hyperglycemia [20]. Another study found that C57BL/6 mice with ovariectomy showed impaired glucose tolerance, that insulin secretion stimulated by glucose from isolated pancreatic islets was significantly reduced, and that supplementation with exogenous estradiol could salvage these effects [21]. Therefore, the glucose variability in women may be related to estrogen levels in men.

**Figure A6:**
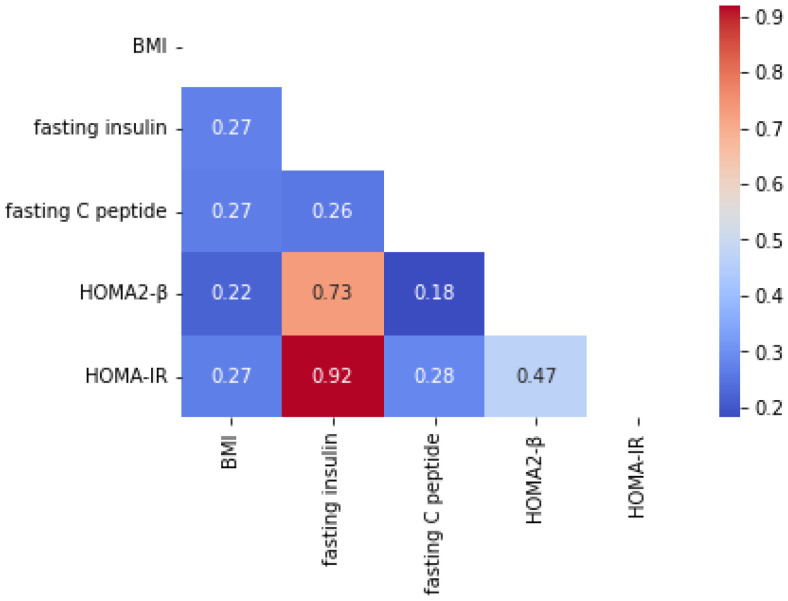
Correlation analysis of BMI with islet function and insulin resistance level HOMA2-β, homeostasis model assessment β-cell function; HOMA IR, homeostasis model assessment insulin resistance.

Our study also found that blood glucose variability of CGM in patients with type 2 diabetes decreased with the increasing BMI, which was consistent with the results of previous studies in non-diabetes and type 2 diabetes population [7,22]. It was found that as BMI increased, pancreatic islet volume [23], insulin secretion [24], and C-peptide content [25] also increased, suggesting that obesity might be associated with better outcomes of β-cell function. However, it was also reported that in diabetes patients the relative islet volume of obese patients was smaller than that of normal weight patients, indicating that in diabetes patients with obesity the β-cell function might not be increased [26]. Therefore, based on the above results, it might be inferred that the reason for the negative association with the glucose variability and BMI may be that obese or overweight individuals might secrete more insulin and also have strong insulin resistance. Moreover, we also supplemented the analysis of the relationship between BMI and pancreatic function and found that BMI was positively related to fasting C-peptide, fasting insulin, HOMA2-β, and HOMA-IR, which indirectly supports this hypothesis (Figure A6).

Moreover, we suggested that the longer duration of diabetes, the higher the glucose variability that was found. However, when multiple linear regression was added, it was age, not the duration of diabetes, that contributed to glycemic variability. Pancreatic β-cell function is acknowledged as a leading factor affecting glucose variability levels in patients with diabetes. Previous studies indicated a negative association between fasting C-peptide and glucose variability in patients with type 2 diabetes [27]. Moreover, they showed that HOMA2-β levels are inversely correlated with glucose variability [28], which is consistent with our findings. A multicenter cross-sectional study further revealed that glucose variability increased while β-cell function declined regardless of the type of diabetes [29]. In addition, due to the lack of data, we are unable to perform the analysis by adopting the M-index, which is a native index of insulin resistance. We look forward to future research exploring the relationship between the M-index and CGM-derived indicators to refine the understanding of these interactions. We found that ALT/AST seemed to be negatively correlated with CV. Although ALT/AST is often associated with metabolic disorders and insulin resistance [30], few studies have directly explored the relationship between ALT/AST and CGM-related data. In addition, we found a positive correlation between HB and HCT with SD, TAR, and eA1c. It was found that HB and HCT were associated with HbA1c, and that anemia may be leading to a decrease in HbA1c concentration [31]; however, no direct evidence has suggested that hemoglobin level was the main factor causing changes in the glucose variability. Further study is needed for this to be verified.

A developed CGMap will be significant for future individualized treatment for patients with T2D. CGM-derived measurements and their relationship to other clinical measurements will serve as tools for researchers and clinicians, with potential applications in areas such as the prediction of the efficacy of hypoglycemic drugs and the long-term outcomes of diet and exercise.

Based data from a multicenter RCT study, a CGMap of T2D has been systematically developed. As CGM devices have gradually been applied to the T2D population, this study might provide new insights to understand the characteristics of CGM devices from populations with T2D, which helps to optimize the management of diabetes for patients with T2D. However, this study still has several limitations. Firstly, only the Chinese population was enrolled in this RCT, and the sample size was relatively limited. The representativeness of the population needs to be improved in future investigations. Secondly, blood glucose fluctuations might also be influenced by the pharmacological interventions and lifestyle modifications. Therefore, the results should be interpreted with caution. Moreover, due to instrument limitations, only 14 days of data can be collected. Therefore, a longer follow-up time is needed in the future to enrich the data points and validate our findings.

## 5. Conclusions

We have successfully developed a CGMap of T2D. It was found that glycemic fluctuations were more frequent and severe in patients with older age, longer duration of diabetes, and lower BMI. Moreover, greater glycemic variability was associated with worse islet function, higher baseline glucose levels, and higher hemoglobin.

## Data Availability

The data that support the findings of this study are available on request from the corresponding author. The data are not publicly available due to privacy or ethical restrictions.

## Abbreviations

The following abbreviations are used in this manuscript:

CGM	Continuous Glucose Monitoring
SMBG	Self-Monitoring Blood Glucose
HbA1c	Hemoglobin A1c
QALYs	Quality-Adjusted Life Years
ADA	American Diabetes Association
T2D	Type 2 Diabetes
MAGE	Mean Amplitude of Glycemic Excursions
SD	Standard Deviation
BMI	Body Mass Index
CV	Coefficient of Variation
GRI	Glycemia Risk Index
RCT	Randomized Clinical Trial
TIR	Time In Range
TBR	Time Below Range
TAR	Time Above Range
eA1c	Estimate Glycated Hemoglobin
FPG	Fasting Plasma Glucose
HOMA2-β	Homeostasis Model Assessment Β-Cell Function
HOMA-IR	Homeostasis Model Assessment Insulin Resistance
SBP	Systemic Blood Pressure
DBP	Diastolic Blood Pressure
SCr	Serum Creatinine
eGFR	Estimated Glomerular Filtration Rate
ALT	Alanine Aminotransferase
AST	Aspartate Aminotransferase
HB	Hemoglobin
HCT	Hematocrit
OLS	Ordinary Least Square

## Appendix A

**Table A1:** Analysis of the Mann–Whitney test in sex.

Group	Sum	GRI	CV	SD	MAGE	TIR	TBR 3.9	TBR 3	TAR 10	TAR 13.9	eA1c
Male	291	14.668 ± 21.632	22.521 ± 6.661	1.660 ± 0.619	4.440 ± 1.800	87.229 ± 18.221	0.456 ± 2.821	0.000 ± 0.373	8.296 ± 17.672	0.246 ± 2.246	6.100 ± 1.175
Female	291	10.298 ± 13.515	22.208 ± 6.443	1.558 ± 0.574	4.190 ± 1.690	90.923 ± 13.048	0.597 ± 2.408	0.000 ± 0.283	6.360 ± 12.055	0.076 ± 0.896	6.000 ± 0.900
Z		−3.341	−0.668	−1.724	−1.694	−1.543	0.471	0.051	−2.086	−2.408	−1.543
P		0.001	0.504	0.085	0.09	0.123	0.638	0.959	0.037	0.016	0.123

Median ± interquartile spacing. GRI, glycemia risk index; CV, coefficient of variation; SD, standard deviation; MAGE, mean amplitude of glycemic excursions; TIR, time in range; TBR, time below range; TAR, time above range; eA1C, BMI, body mass index.
